# SGC - Structural Biology and Human Health: A New Approach to Publishing Structural Biology Results

**DOI:** 10.1371/journal.pone.0007675

**Published:** 2009-10-20

**Authors:** Wen Hwa Lee, Julián Atienza-Herrero, Ruben Abagyan, Brian D. Marsden

**Affiliations:** 1 SGC, University of Oxford, Headington, Oxford, United Kingdom; 2 Skaggs School of Pharmacy and Pharmaceutical Sciences, University of California San Diego, La Jolla, California, United States of America; 3 Molsoft LLC, La Jolla, San Diego, California, United States of America; 4 Nuffield Department of Clinical Medicine University of Oxford, Headington, Oxford, United Kingdom; University of Cambridge, United Kingdom

The Structural Genomics Consortium (SGC) is a not-for-profit, public-private partnership established to deliver novel structural biology knowledge on proteins of medical relevance and place this information into the public domain without restriction, spearheading the concept of “Open-Source Science” to enable drug discovery. The SGC is a major provider of structural information focussed on proteins related to human health, contributing 20.5% of novel structures released by the PDB in 2008. In this article we describe the PLoS ONE Collection entitled ‘Structural Biology and Human Health: Medically Relevant Proteins from the SGC’. This Collection contains a series of articles documenting many of the novel protein structures determined by the SGC and work to further characterise their function. Each article in this Collection can be read in an enhanced version where we have integrated our interactive and intuitive 3D visualisation platform, known as iSee. This publishing platform enables the communication of complex structural biology and related data to a wide audience of non-structural biologists. With the use of iSee as the first example of an interactive and intuitive 3D document publication method as part of PLoS ONE, we are pushing the boundaries of structural biology data delivery and peer-review. Our strong desire is that this step forward will encourage others to consider the need for publication of three dimensional and associated data in a similar manner.

## Introduction

Since its advent, structural biology has played a major role in the understanding of biological processes at the molecular level. This, in turn, has enabled the progression and development of several fields in biological sciences. As a consequence there has been a continuing demand for faster and more cost-effective determination of protein structures and their in-depth functional, mechanistic and biological analysis. The lack of a well-established process historically meant that structures were been solved in a case-by-case manner, with academic researchers establishing specific protocols over a relatively long period of time that are difficult to implement or inappropriate for high-throughput approaches. As the sequencing of the human genome was completed the gap between known genes and solved protein structures became wider. As a result concerted and systematic technology-driven ‘structural genomics’ (SG) efforts to solve protein structures in a high throughput manner were begun (for thorough reviews see [1], [2]).

First formalised in 1998 [3]–[5], several major SG efforts were subsequently started such as the NIH-funded Protein Structure Initiative (PSI; http://www.nigms.nih.gov/Initiatives/PSI), European initiatives ‘The Protein Structure Factory (PSF; http://www.proteinstrukturfabrik.de) and ‘Structural Proteomics in Europe (SPINE; http://www.spineurope.org) and Japanese Protein 3000 and RIKEN Structural Genomics/Proteomics programs (http://protein.gsc.riken.jp). The majority of these early programmes were tasked with exploring the breadth of the protein structural universe by focussing on prokaryotic targets whilst driving novel structural biology technology for a high-throughput environment [1], [6].

New avenues have been opened by the availability of detailed structural descriptions of proteins and enzymes involved in the normal (or abnormal) functioning of the human body and associated pathogens. In particular, this information provides a better understanding of the basis by which different proteins achieve selectivity and specific binding to their cognate ligands and drugs [1]. Indeed, several marketed drugs in recent years have been developed using structure-based methods, targeting a wide range of disease such as AIDS, leukaemia, cancers and venous thromboembolic events [1], [7]–[10].

Despite these successes, determining the structures of human proteins in a high-throughput manner was seen to be technically challenging relative to those of less complex organisms (e.g. prokaryotes), where issues such as the lack of solubility of expressed proteins in simple bacterial systems pose major bottlenecks to scaling the structure determination process [11]. The SGC (http://www.thesgc.org) was created in 2003 as a response to this challenge.

The SGC is a not-for-profit organisation that aims to solve the three dimensional structures of proteins of medical relevance and place them into the public domain without encumbrance or restriction. The SGC has adopted a protein-family based approach whereby protein targets are chosen from discrete and medically-relevant human protein families. This approach ensures that comparative analysis and “blanket” methods can be applied to members of same family [11]. The SGC is driving the concept of “open-source science” to enable drug discovery by promoting pre-competitive structural biology and medicinal chemistry. This is achieved by combining academic and industrial efforts to create open-access chemical and clinical probes [12]–[14] which might be used in a pre-competitive manner for target validation purposes, for example.

Since the production phase of the SGC began in 2004, we have released the coordinates of over 800 protein structures related to several diseases and metabolic disorders into the public domain of which over 700 are novel and unique. Whilst the SGC focuses primarily on human targets, we also pursue the structure determination of proteins from parasitic organisms associated with neglected diseases, such as malaria and toxoplasmosis. We have deposited more than 70 of these structures into the public domain. These structures are providing a significant impact with the SGC providing 61% of publicly available apicomplexan structures. The SGC has also developed new protocols and methods that are now widely used [15]–[18] and is developing chemical probes [19] and small molecules in areas such as human epigenetics and human protein kinases, to be placed in the public domain [1] as part of the ‘open-source’ science approach.

In this PLoS ONE Collection we present articles covering the structure determination of a number of targets by the SGC along with additional work carried out to further characterise these proteins. Since SG data is intrinsically heterogeneous and not always accessible to non-specialists, all the articles from this collection are available also as an enhanced version using our interactive visualisation platform – known as iSee – which allows the reader to interact and manipulate the 3D scenes prepared by the authors to highlight and support information given in the main text of the article [20].

## SGC and iSee: extending the delivery of structural biology data

A clear mandate of the SGC is to make results readily available to the scientific community. This data is necessarily heterogeneous in nature, ranging from materials and methods, construct and clone design, protein expression and purification to small molecule screening and protein structure determination information. The primary ‘customers’ for this information are not necessarily structural biologists but scientists with little if any structural biology understanding such as medicinal chemists, clinicians, pathologists etc.

Historically, SG efforts have solved structures of proteins with unknown function, relying on the scientific community to take these proteins, characterise them annotate the structures. Since the structures released by the SGC are of targets with known biochemical and, often, biological function, it is possible to interpret the structures in these contexts thereby providing new insights into the role of the targets.

However, the deposition into specialist databases such as the Protein Data Bank of the protein structures is not sufficient to reach the ‘customers’ of this data. There are a number of significant challenges that face an interested scientist who is not a structural biologist. Firstly, the scientist must be aware of where and how a protein structure can be retrieved. Secondly, it is necessary to visualise the structure using software that may not be intuitive to a non structural-biologist. Thirdly, the interpretation of the structure is dependent not only on an understanding of the biology and function of the protein, but also the structure itself. Alternatively, protein structures may be published in peer-reviewed journals. However, this medium often suffers from stringent restrictions in size by the publishers, forcing the authors to use jargon and cutting down the elementary information needed for the comprehension by non-structural biologist.

Representing a 3D protein structure using media that is in reality 2D also poses an obstacle. The visualisation of protein structure on a computer screen is a clear improvement over a paper figure, even if stereo images are provided. Several movie-making tools (e.g. PMG [21], eMovie [22], VMD [23], MovieMaker [24], POLYVIEW-3D [25], YASARA movie (http://www.yasara.org)) are an alternative to convey a sense of depth and perspective to structures. However, this approach provides ‘fixed’ set of visualisations as defined by the movie authors resulting the viewer being unable to interact with the structure displayed. This hurdle is overcome using specialist visualisation software (e.g. PyMol (http://www.pymol.org), Jmol (http://jmol.sourceforge.net), Kinemage/KiNG [26], [27], Swiss PDB Viewer [28], FirstGlance (http://molvis.sdsc.edu/fgij), RCSB Protein Workshop [29]). Some of these platforms are feature-rich from the perspective of a structural biologist making them somewhat inaccessible and unintuitive to use by others. Others are simple to use but at the cost of functionality and the ability to replicate, for example, figures produced in peer-reviewed articles.

The SGC is unable to write and publish papers on every structure we solve but yet needs to provide results to the widest audience possible. Given this, we recognised that an intuitive solution to the aforementioned issues would be to combine expert-written explanatory text with relevant portions linked to pre-staged scenes, using the underlying data, to illustrate the points being made. Importantly, these pre-staged views should be able to be invoked in any order in a seamless fashion and allow the reader to manipulate the visualisation as they see fit.

The resulting platform is known as iSee was launched in 2005. iSee integrates key data from all stages of the high throughput structural biology pipeline, from cloning details and molecular biology/protein chemistry protocols to the full coordinates of the solved structures. The structural information is discussed, visualised and annotated by experts of the protein families within the SGC, focussing on making the results as accessible as possible to a wide range of readers [20].

**Figure 1:**
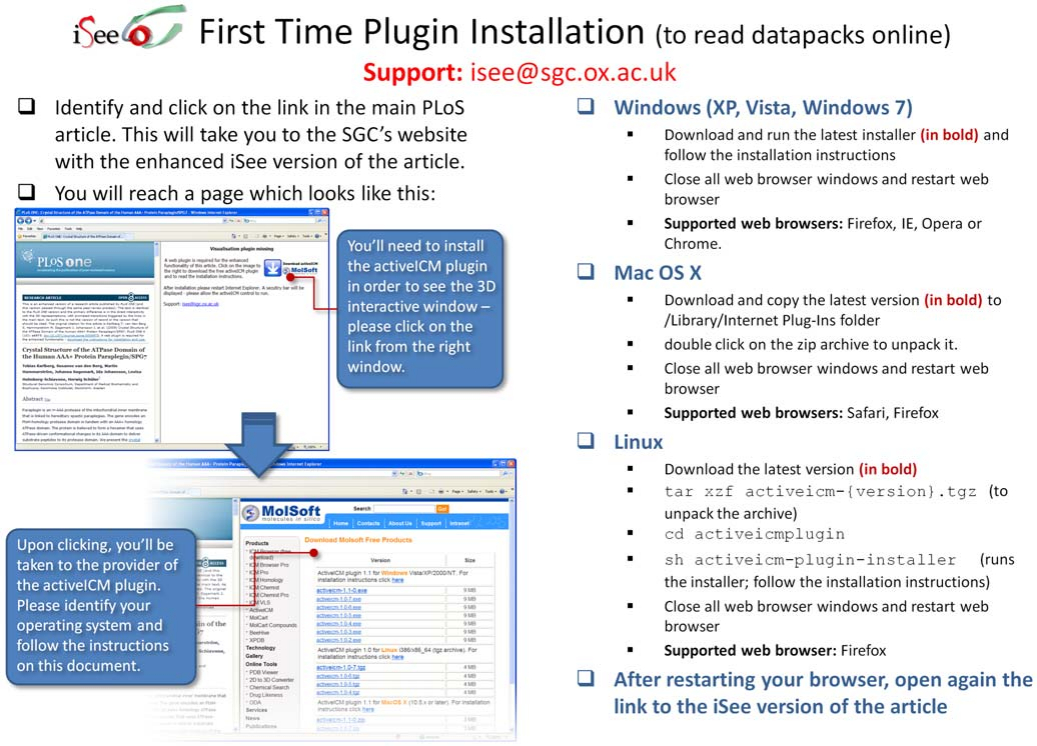
Reading the iSee version of the articles on-line - first-time installation instructions.

**Figure 2:**
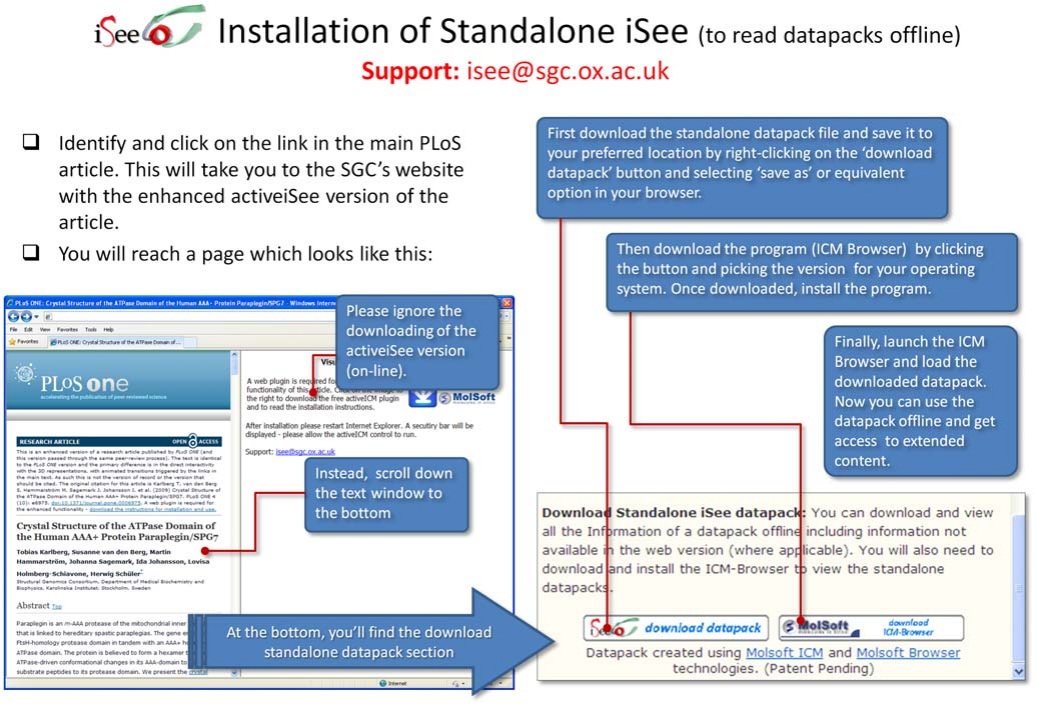
Reading the iSee version of the articles offline - first-time installation instructions.

The iSee concept was implemented using Molsoft's ICM platform [30], as this was the most mature platform available for this approach at the time. ICM is used to bundle a large number of heterogeneous data types together as a single, small computer file (please refer to the 3D document overview article by Abagyan *et al.* in this Collection for more details) and provide high quality graphical visualisations of that data. Each file – which we dub an ‘iSee datapack’ – is usually smaller than 10 Mb and can be downloaded freely from the SGC website (www.thesgc.org/iSee). To open these datapacks online, a free plugin is available for the most common internet browsers and platforms such as Windows, Linux and Mac OS/X with Internet Explorer, Mozilla Firefox, Google Chrome and Safari (Figure 1). An offline standalone free reader is also available, known as ICM Browser (Figure 2).

**Figure 3:**
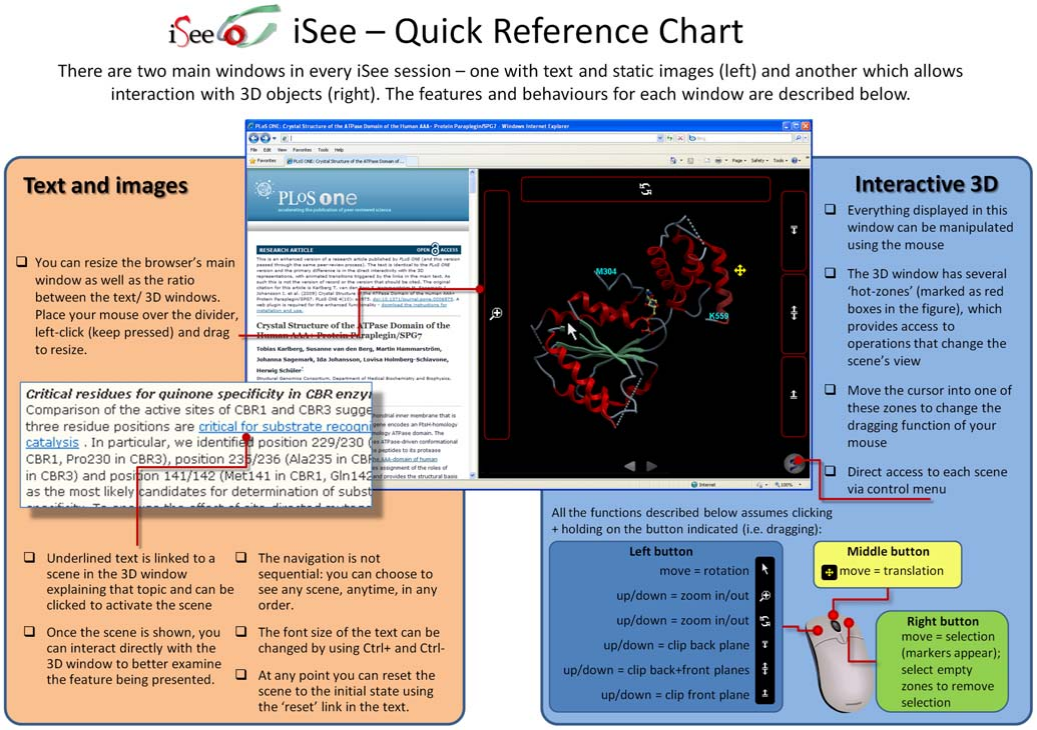
Main features of the enhanced iSee version of the articles and a quick reference guide to interact and use the graphical display window. Both on-line and offline (standalone) versions of the enhanced iSee articles have the same controls and layouts.

A datapack is made up of two major interface components: an annotation text pane corresponding to the textual element of a paper, and a graphical pane where high-quality 3D visualisations are shown (Figure 3). Within the text words or phrases are linked, in the style of web hyperlinks, to the triggering of an animation or transition to a pre-programmed scene in the graphical pane depicting important structural features which are discussed in the text. Each of these scenes (or 3D slides, as they are known) is prepared by the datapack's author in conjunction with the text. Since there is no physical space limit on the number of these slides, the datapacks have significantly more ‘figures’ embedded than is generally found in traditional papers. Furthermore, these Figures are only the starting points for readers who are then able to interactively adjust the view and capture its three dimensional complexity. Successive clicks on different links trigger animated transitions, which are computed ‘on-the-fly’, from one scene to another, helping the reader to maintain context within in the structure and establish the spatial relationship between features. Importantly, the ability to calculate transitions upon request means that the reader has direct control over the flow of reading as the links can be clicked on in any order desired. All these features are implemented in the program ICM (www.molsoft.com; [30]), in a user-friendly way, to enable scientists outside SGC to easily create their own iSee-like datapacks.

Each SGC iSee datapack is target-centric, making datapacks a one-stop shop for SGC data for a given target. Where there are multiple structures for a target determined by the SGC, often with different co-complexed ligands, these are included and frequently compared. Since 2005 the SGC has authored more than 500 datapacks covering more than 600 structures deposited by the SGC. At the time of writing, there have been more than 25,000 unique downloads of SGC iSee datapacks.

Since the initial launch of the iSee concept, several implementations along similar lines have been launched. For instance Nature Structural Biology has adopted a simple linking to an external 3D visualiser for articles with structures and associated PDB codes [31].

The Proteopedia initiative [32] employs the core of the iSee concept, whereby selected portions of an explanatory text are linked to interactive scenes. It is entirely web-based, employing Jmol as the viewing engine. Its key innovation lies in the adoption of a community-authored Wiki model. This allows a readers to find structures deposited at the PDB and author annotations to capture key scientific information about the protein.

In September 2008 Kumar *et al.* [33] proposed and demonstrated the embedding and display of 3D objects within a PDF file. For the first time in a structural biology publication they utilised a pre-existing feature of the Adobe Acrobat package that has been little-known to the biomedical community (but commonly used by CAD/engineering communities). Unfortunately, the process to produce embedded 3D objects within a PDF is non-trivial in the context of structural biology data and so far (to our knowledge) only the author's article has been published using this method. Moreover all the 3D objects using this format are merely described as surfaces and do not retain any of the underlying scientific information from the original structural data, making it suitable for simple viewing only. This is because the use of surfaces means that, apart from colour changes and viewpoints, every new interactive scene needs to be created as novel surfaces, at the expense of file size.

Recently publishers have indicated their awareness and desire to explore this field further. The initiative from Cell Press and Elsevier entitled ‘Article of the Future’ (http://beta.cell.com) explores the advantages of various modes in which a web-based publication of the future may be delivered. The Journal of Biological Chemistry also encourages the use of 3D PDFs to communicate structural information [34]. We welcome these initiatives and expect that more will come to light as the demands of authors and readerships change, aligning closely with our iSee concept.

## The PLoS ONE Collection: peer-reviewing iSee datapacks

The PLoS ONE Collection, entitled ‘Structural Biology and Human Health: Medically Relevant Proteins from the SGC’ contains a continually expanding series of articles documenting many of the novel protein structures determined by the SGC and work to further characterise their function. In addition to the standard PLoS ONE online delivery mechanism, each article is available as an online iSee datapack, enabling the delivery of our data in an interactive and intuitive 3D manner.

An important aspect of this PLoS ONE Collection is that, for the first time in an open-access journal, the peer-review process of these articles is carried out upon the enhanced non-paper based iSee datapack versions themselves. We believe that this allows reviewers to take advantage of the information integrated in a single file, in addition to the 3D data including live 2D data attachments such as chemical or standard spreadsheets and plots, alignments, etc. Additionally, this also saves the referees' time in that there is no immediate need to retrieve, analyse and validate the structural data, for these are all included in the iSee datapack itself.

We have also made the iSee version of our articles open-access, using the Creative Commons Attribution license in common with all PLoS ONE articles, ensuring that the full content can be further extended if any reader wishes to do so. This allows other organisations to capture and even extend the information published in the articles and integrate it into novel platforms.

Further discussion and feedback of our articles is encouraged and enabled on the PLoS ONE website. Both the HTML/PDF and the enhanced iSee versions will remain constant in terms of content and location. This is critically important for future reference and also archival purposes. For technical questions and feedback we have implemented an iSee-Wiki (http://www.thesgc.org/iSee/wiki) maintained by specialists to address the most common questions and to provide usage and authoring tips.

## Three-dimensional documents - the future

Currently, iSee datapacks are provided in a binary file format to enable maximal compactness and fast download to the browser. A full text file format would increase the files' size resulting in relatively slow downloads into the browser/plugin. However we strongly believe that it is vital that we and others are able to provide similar data and its interpretation in a completely open manner. Not only is this important for the ability to curate and archive such work, it is also critical to allow the data to be opened and displayed in other visualisation platforms. To enable the portable delivery of such content we are working with the structural biology visualisation community to begin work on an open file format which can provide a human readable and editable version of any set of data, visualisations and annotations.

## Conclusion

We are excited to be making the SGC's work available to a greater audience via this PLoS ONE collection. With the use of iSee as the first example of an interactive and intuitive 3D document publication method as part of PLoS ONE, we are pushing the boundaries of structural biology data delivery and peer-review. Our strong hope is that this step forward will encourage other publishing houses to consider the need for publication of three dimensional data in a similar manner.
